# Dihydrofolate reductase as a model for studies of enzyme dynamics and catalysis

**DOI:** 10.12688/f1000research.6968.1

**Published:** 2015-12-17

**Authors:** Amnon Kohen

**Affiliations:** 1 Department of Chemistry, University of Iowa, Iowa City, IA, USA

**Keywords:** Dihydrofolate reductase, Escherichia coli, catalysis

## Abstract

Dihydrofolate reductase from *Escherichia coli* (ecDHFR) serves as a model system for investigating the role of protein dynamics in enzyme catalysis. We discuss calculations predicting a network of dynamic motions that is coupled to the chemical step catalyzed by this enzyme. Kinetic studies testing these predictions are presented, and their potential use in better understanding the role of these dynamics in enzyme catalysis is considered. The cumulative results implicate motions across the entire protein in catalysis.

## Introduction

Enzymes are large and flexible proteins that catalyze most chemical reactions in life. The dynamics of a protein's folding from a linear polymer to the globular and active form of the enzyme are rather well understood. The role of the motions and vibrations of the folded enzyme throughout its catalytic cycle, on the other hand, is a matter of intensive investigation. Here we will present studies of *E. coli* dihydrofolate reductase, or DHFR, that focus on the role of dynamics across the protein in the chemical step catalyzed by this enzyme. DHFR from *E. coli* is a preferred model system for such studies because it is a small monomeric enzyme, has no metals or S-S bonds, and folds reversibly. Additionally, as this DHFR has been used in many diverse studies, the body of available information on this enzyme opens the door to in-depth physical studies such as those presented below.

We must first address some controversy that has arisen in the field regarding terminology. The terms “dynamics” and “catalysis” are defined differently by different researchers. Most biochemists and several chemists use the term “dynamics” to refer to *any* vibration or motion of the protein complex with its ligands and solvent. That definition includes statistical and non-statistical dynamics (motions and vibrations). By contrast, several physical chemists define the term “dynamics” more narrowly, using it to refer only to chemical, or non-statistical, dynamics (motions or vibrations that are *not* in thermal equilibrium with their environment). As for the term “catalysis”, it is formally defined as the ratio of catalyzed and uncatalyzed turnover rate constants under the same conditions. Unfortunately, performing studies of the uncatalyzed reactions is frequently challenging or even unrealistic for both experimentalists and theoreticians. Computer-based calculations that have attempted to compare catalyzed to uncatalyzed reactions have usually begun with the enzymatic reactive complex, substituted water for the protein, and restricted the reactants to the orientation found in the enzyme in order to calculate the uncatalyzed reaction. However, in reality, the statistics of bringing reactants to the reactive orientation in question do not agree with the calculations, and some of these uncatalyzed reactions never actually occur experimentally without the catalyst. Consequently, most studies of “enzyme catalysis” address only the enzyme-catalyzed reaction, not the comparison to the uncatalyzed reaction. Since these researchers (probably a majority) still use the term “catalysis” (rather than “enzyme-catalyzed reaction”), they and their titles are condemned by some who assume they refer to the comparison with the uncatalyzed reaction. In reality, very few experimental studies have compared the catalyzed to the uncatalyzed reactions ^1^, and (as far as I am aware) no experiment today can distinguish between statistical and non-statistical dynamics in an enzyme-catalyzed reaction ^2^.

Returning to ecDHFR, to the best of my knowledge, no relevant uncatalyzed reaction has yet been reported for this enzyme. Since non-statistical dynamics cannot be tested on their own experimentally, no such dynamics are proposed below when addressing protein dynamics that participate in catalysis. Instead we use a thermally equilibrated model that seems to be in accordance with all experimental findings ^2^, and “dynamics” here will mean all vibrations or motions in the protein complex (including solvent and ligands).

## Studies of dihydrofolate reductase

**Figure 1.**
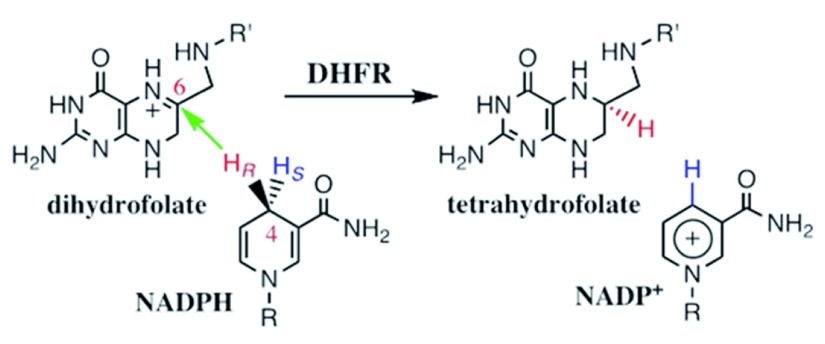
Dihydrofolate reductase catalyzed C-H→C hydride transfer. The reaction catalyzed by dihydrofolate reductase (DHFR). R = adenine dinucleotide 2’ phosphate and R’ = (p-aminobenzoyl) glutamate. It has been shown that the protonation of the N5 position of DHF occurs prior to hydride transfer, at all relevant media pH (5–11.5) ^3^.

**Figure 2.**
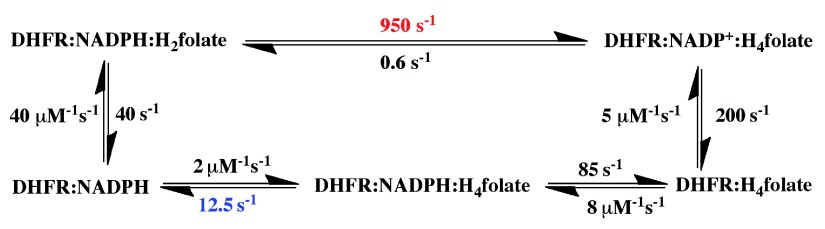
The catalytic cycle of *Escherichia coli* dihydrofolate reductase. During its turnover, *Escherichia coli* dihydrofolate reductase (ecDHFR) cycles through 5 kinetic intermediates. The rate constants of all steps are from 4. The pH-independent rate (950 s ^-1^ in red), sometimes addressed as the hydride-transfer rate, was obtained from non-linear regression of the pH dependence of observed single-turnover rate constants ^4^. The overall rate-limiting step on the catalytic turnover number, *k*_cat_, is 12.5 s ^-1^ (in blue).

Mapping a network of enzyme-wide motions involved in catalyzing the chemical conversion requires the ability to experimentally probe the chemical step within the enzyme’s complex kinetic cascade. The chemical step catalyzed by DHFR is a C–H→C hydride transfer, shown in Figure 1. The enzyme catalyzes the NADPH-dependent reduction of 7,8-dihydrofolate (H _2_folate) to 5,6,7,8-tetrahydrofolate (H _4_folate), which is the reactive form of folic acid, and is a critical one-carbon carrier in DNA nucleotides’ biosynthesis and other cellular processes. It has been shown that N5 of H _2_folate is protonated by the enzyme prior to the hydride transfer step ^3^. This fact greatly simplifies the calculations and data interpretation for this enzyme, as these can focus on a single barrier event. This fact, however, makes the assessment of the experimental rate constant of the C–H→C hydride transfer very challenging. While computer-based molecular calculations address only that chemical step, representing a single kinetic barrier, the rate for that single step is quite impossible to assess experimentally, leaving little room for direct examination of the theoretical predictions. To emphasize this last point, Figure 2 presents a minimal kinetic scheme for this enzyme ^4^. As one can see in this scheme, most of the enzyme is never free. The release of the first product (NADP ^+^) is followed by the binding of the substrate NADPH, and only then is the product H _4_folate released, prior to the binding of the second substrate, H _2_folate, to form the reactive complex. This scheme indicates that steady-state kinetic parameters (i.e., *k*_cat_ and *k*_cat_/ *K*_M_) do not always reflect the chemical step, which can be much faster than other kinetic steps.

To handle this type of problem it is usual to employ pre-steady-state kinetics, in which the substrate under investigation (e.g., NADPH) is pre-bound to the enzyme, and the reaction is initiated by a very high concentration of the second substrate (in this case H _2_folate). The conversion of NADPH to NADP ^+^ on the enzyme is followed spectroscopically, so neither substrate binding nor product release affects the rate constant. However, the second substrate, H _2_folate, is not protonated in solution (at physiological pH). Thus, after its very fast binding to the enzyme-NADPH complex, major changes in the active site are required (involving at least residues D27 and Y100 ^3^) in order to protonate this substrate prior to the hydride transfer, and bring it into the reactive conformation in the ternary complex. This problem with using pre-steady-state rate constants is also apparent from the pH-dependence of these observed rate constants ^4, 5^. The only way to assess a pH-independent pre-steady-state rate constant is by measuring the pH dependence across a broad pH range, and extrapolating to infinitely low pH ^4^. Unfortunately, this has been a common practice only in Benkovic’s lab ^4, 6^.

Figure 2 also makes plain another, more serious problem with the pre-steady-state approach for ecDHFR: the measured pre-steady-state rate constant, even when extrapolated to a low pH, is at the millisecond timescale (e.g., 950 s ^-1^ in Figure 2). This rate constant is much slower than the C–H→C hydride transfer *per se*, which takes place at the picosecond to femtosecond timescale.

**Figure 3.**
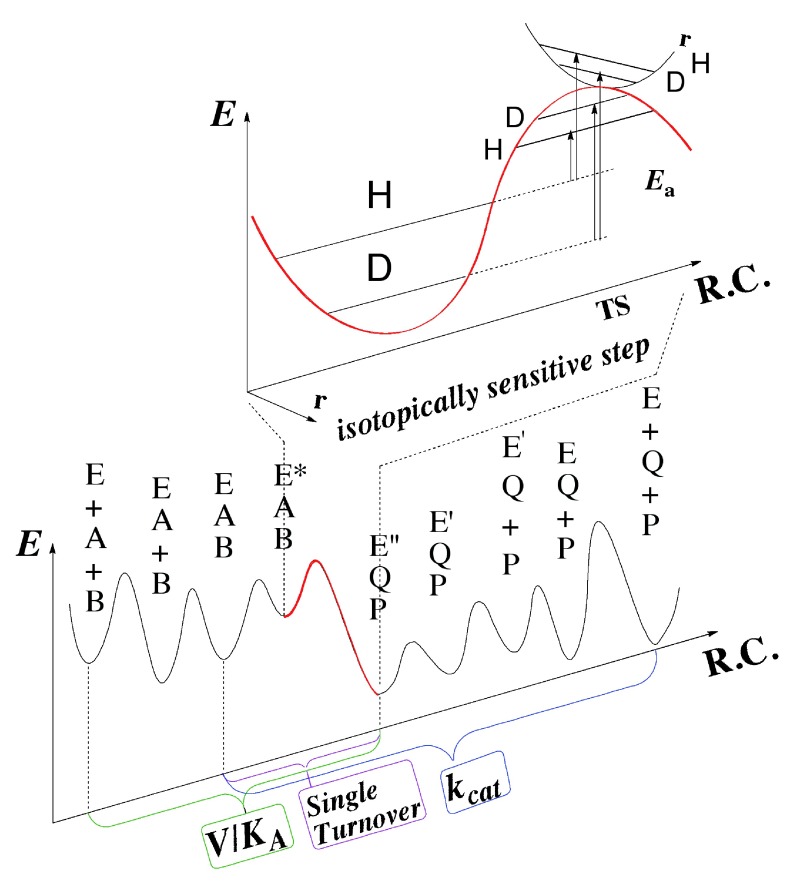
An illustration of the difficulty in assessing the rate of the chemical step (red) catalyzed by an enzyme. Both steady-state parameters (e.g., *k*_cat_/ *K*_M_ (V/K) and *k*_cat_) and pre-steady-state rates (e.g., single-turnover rate) involve several microscopic rate constants, which may not represent the rate of the chemical step. In *Escherichia coli* dihydrofolate reductase (ecDHFR), pH 7, *k*_cat_ mostly represents the release of the product H _4_folate, *k*_cat_/ *K*_M_ mostly represents the binding of the substrate H _2_folate ^4^, and the single-turnover rate mostly represents the conversion of the initial ternary complex (Enz.NADPH.H _2_folate) to its reactive form with protonated-H _2_folate ^3^. The intrinsic kinetic isotope effects (KIEs) (whether resulting from differences in zero-point-energy or from quantum tunneling or both) better reflect the chemical step *per se*, but assessing these from their observed values is quite challenging (see text).

A more direct experimental method of investigating only the chemical step is to study kinetic isotope effects (KIEs), comparing the rates or rate constants of two substrates in which the cleaved C-H bond carries different isotopes (e.g., H/D, H/T, or D/T). In such a study, the complex kinetic expression that constitutes the absolute rate is greatly simplified, as many steps that are not isotopically sensitive (i.e., steps that do not include the C-H cleavage in this case) fall out of the KIE equation (ratio of rates). This point is emphasized by Figure 3, which indicates how difficult it is to determine rate constants on the H-transfer step from either steady-state kinetics (e.g., *k*_cat_ or *k*_cat_/ *K*_M_) or pre-steady-state kinetics. The scheme shows that steps other than the C–H→C hydride transfer under study are frequently rate-limiting for the kinetic parameter measured. Kinetic isotope effects (KIEs) are also not complexity-free, and the observed KIEs are often smaller than their intrinsic value on the chemical conversion itself ^7, 8^. This said, KIEs are a useful way to examine the chemical step when a method is used to assess their intrinsic value ^9– 11^.

As illustrated in Figure 3, most of the KIE results from differences between the zero point energies (ZPE) of the ground state and the transition state, and can be also affected by nuclear-quantum-mechanical tunneling (a phenomenon in which the atom is transferred under the classical energy barrier via its wave-like properties). In the case of H-tunneling, the transition state becomes the tunnelingready state (TRS), or the chemically reactive state, which is the quantum mechanically delocalized transition state ^2^. Importantly, the intrinsic KIE is an experimentally measurable ratio of rates that can be directly compared to molecular calculations of the chemical step *per se*.

To better assess the intrinsic KIEs for DHFR, we used all three isotopes of hydrogen (i.e., the Northrop-method) ^12, 13^. In contrast to most other methods, this method makes assumptions that might slightly affect the size of the KIE, but are not likely to alter its temperature dependence. This is useful because the temperature dependence of intrinsic KIEs is a sensitive probe of the nature of the H-transfer or, more specifically, the donor-acceptor distance (DAD) dynamics and distribution at the TRS ^2, 14, 15^. It is fair to say that the temperature dependence of KIEs is a more meaningful probe of the nature of the catalyzed chemical step than are the rate constants or KIEs themselves ^2^.

In most wild-type and well evolved enzymes, it has been found that the intrinsic KIEs for H-transfer reactions are temperature-independent ^14^, suggesting the enzymes evolved to have short and narrowly distributed DADs (i.e., a well- reorganized TRS) ^16^. Mutations that affect the chemical step, as well as unnatural substrates or non-physiological reaction conditions, often lead to more highly temperature-dependent KIEs (with poorly reorganized TRSs). The broader distribution of DADs means lower frequency of DAD sampling at the TRS, and this dynamic search for short DADs results in increased KIE temperature dependence.

## Experimental test of computed prediction

**Figure 4.**
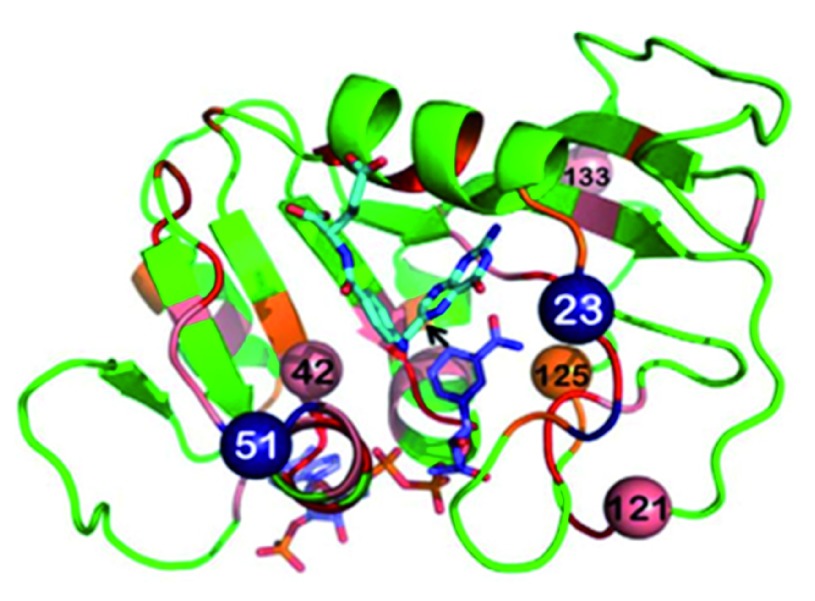
Structure of dihydrofolate reductase from *Escherichia coli* highlighting the residues under study. Dihydrofolate reductase (DHFR) structure (PDB ID 1rx2; 18) colored based on genetic coupling analysis as conserved (red), strongly coupled (pink), and weakly coupled (orange). The NADPH cofactor (dark blue) and folate (light blue) are highlighted as sticks, and an arrow is drawn at the path of the hydride transfer under study. The four coevolving residues that are discussed in the text are highlighted as spheres, and the insertion sites at N23 and G51 are highlighted as dark blue spheres. Reproduced from 15 with permission from the American Society of Biochemistry and Molecular Biology.

A test case is presented below in which the temperature dependence of intrinsic KIEs was used to study a predicted network of coupled motions in the DHFR from *E. coli* ^17^. Figure 4 presents residues that are predicted to be coupled to each other and be part of the reaction coordinate of the C–H→C hydride transfer steps. Some of these residues are in the active site and in direct contact with the reactants (e.g., I14), while others are far from the active site, and their coupling to the chemistry catalyzed by the active site is not trivial at all.

**Figure 5.**
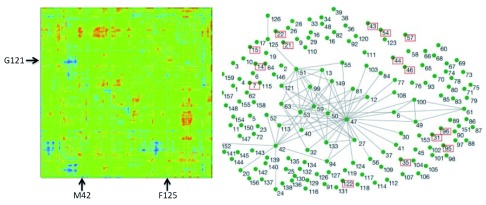
Coupling predicted from QM/MM and bioinformatics calculations. Left Panel: QM/MM calculations. A map of all *Escherichia coli* dihydrofolate reductase (ecDHFR) residues that are coupled to each other and to the reaction coordinate. The two axes are identical and represent the atoms of the enzyme in sequential order. Residues under study here are marked by arrows (M42, G121, F125). Right Panel: Bioinformatics calculations. The evolutionary coupling network of amino acid residues in DHFR, with highly conserved residues in red boxes and lines connecting residues that co-evolve (i.e., are genetically coupled). Reprinted from 21 and 22 with permission from National Academy of Science and Annual Reviews, respectively.

Various molecular calculations of DHFR from *E. coli*, along with bioinformatics statistics of DHFR from various organisms, predicted that several residues in the enzyme’s active site as well as several residues remote from the active site are dynamically and genetically coupled to the catalyzed reaction ^19– 23^. The term genetically coupled refers to residues that co-evolve, and thus while they are not highly conserved, their distribution differs from random statistical distribution ^22^. One example from a molecular calculation and one from bioinformatics are presented in Figure 5. Interestingly, both methods predicted that several residues are coupled to the chemistry (e.g., G121, M42, and F125), while others were implicated only by bioinformatics (e.g., W133). To test these predictions, we measured the intrinsic KIEs for the wild-type enzyme and for its single and double mutants, testing residues predicted to be coupled by one of these methods or both.

**Figure 6.**
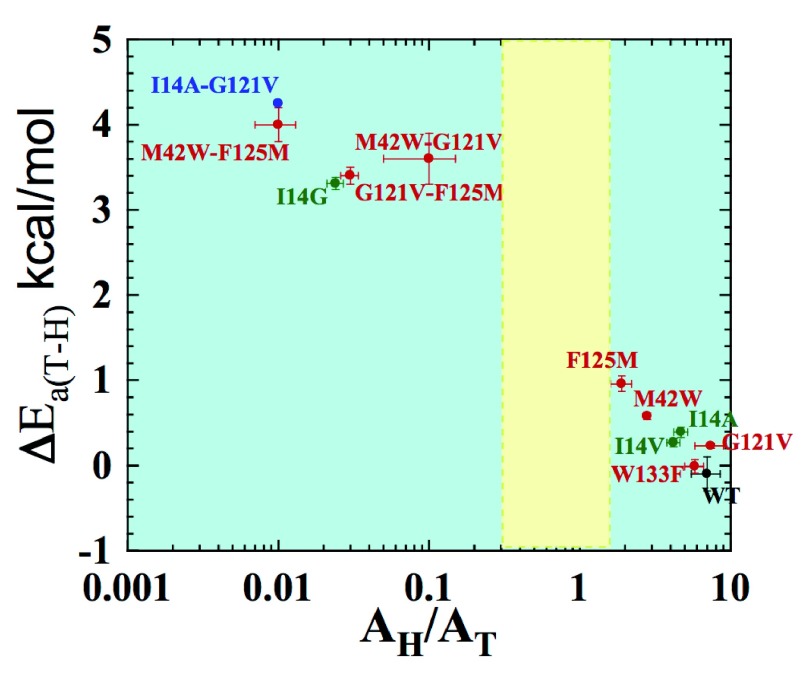
Correlation chart of the isotope effects on the activation parameters. Presented are data for wild-type (WT) (black), distal (red), and local (green) mutants of *Escherichia coli* dihydrofolate reductase (ecDHFR). The double mutant bridging local and distal is in blue. Error bars represent standard deviation. The yellow block represents the semi-classical range of the Arrhenius pre-exponential factor (0.3–1.7). Reproduced from 26 with permission of the American Chemical Society.

Figure 6 summarizes the isotope effects on the activation parameters for the intrinsic KIEs, where Δ *E*_a (H/T)_ represents the slope of the temperature dependence of the H/T KIEs (the larger the value, the more temperature-dependent are the KIEs), and A _H_/A _T_ is the isotope effect on the Arrhenius pre-exponential factors. The green data points are for single mutations at the active site (I14 to V, A, and G), each of which is designed to generate a poorly reorganized TRS by decreasing the size of an enzymatic side-chain holding the H-donor close to the acceptor ^24^. For these mutants, the smaller the side chain is, the larger is Δ *E*_a (H/T)_ and smaller A _H_/A _T_. This observation is in accordance with the fact that longer DADs, with broader distributions, are associated with greater temperature dependence of intrinsic KIEs. The effect of active site mutations on the catalyzed reaction and its DAD is more obvious than that of remote mutants. Studies of mutants far from the active site indicate that double mutants have a non-additive effect that is much larger than their respective single mutants (Δ *E*_a (H/T) double mutant A&B_ > Δ *E*_a (H/T) mutant A +_Δ *E*_a (H/T) mutant B_). This finding supports the prediction that those residues are coupled to each other along the reaction coordinate for the hydride transfer in the wild-type enzyme. Interestingly, I14 is also found to be coupled to remote residues like G121, offering a path through which the remote residues can affect the H-transfer step ^25^.

In accordance with predictions made by the calculations presented above, these findings indicate that several residues across the whole protein, including some far from the active site, are involved in a network of coupled motions that affect the chemical step catalyzed by DHFR from *E. coli*. The fact that the bioinformatics calculation alone predicted that W133 is coupled, yet it had no visible effect on the chemical step, suggests that there might be more than one functional network. In addition to a network that affects activation of the chemical bond, there could be networks that are important for proper folding or for other biological functions. Notably, a very different type of calculation did not predict “protein promoting vibrations” to be part of the chemical step in this enzyme ^27^, but it is not clear that these calculated vibrations address the same phenomena and motions on the same timescale as those examined by the studies ^19– 23^ and experiments presented above ^24– 26, 28^.

## Concluding remarks

An important take-home message from the above studies is that observed rate constants and KIEs should not be taken as a probe for a single kinetic step. These observed values often represent a complex kinetic expression rather than the chemical step *per se*. The observed rate constants and KIEs, and especially their temperature dependence (and thus their activation parameters) often reflect complex phenomena involving many microscopic rate constants. This is unfortunate, as most calculations address only the barrier that is the chemical step, but not other steps affecting the experimental measurement. In many cases the bond cleavage step of interest occurs at the picosecond to femtosecond timescale and is a fleeting event within catalytic turnover, which occurs at the millisecond timescale. Not many experimental methods are available that report on the time scale of the bond activation, yet the ability to probe intrinsic parameters that probe the chemical step, such as those presented in Figure 6, is critical when experimentally assessing any molecular calculation that focuses on the single kinetic step in which the chemical conversion occurs.
